# A phase 1b dose escalation study of Wnt pathway inhibitor vantictumab in combination with nab-paclitaxel and gemcitabine in patients with previously untreated metastatic pancreatic cancer

**DOI:** 10.1007/s10637-019-00824-1

**Published:** 2019-07-23

**Authors:** S. Lindsey Davis, Dana B. Cardin, Safi Shahda, Heinz-Josef Lenz, Efrat Dotan, Bert H. O’Neil, Ann M. Kapoun, Robert J. Stagg, Jordan Berlin, Wells A. Messersmith, Steven J. Cohen

**Affiliations:** 1 University of Colorado Cancer Center, Aurora, CO USA; 2 Vanderbilt University, Nashville, TN USA; 3 Indiana University, Indianapolis, IN USA; 4 University of Southern California, Los Angeles, CA USA; 5 Fox Chase Cancer Center, Philadelphia, PA USA; 6 OncoMed Pharmaceuticals, Redwood City, CA USA; 7 Jefferson Health/Abington Memorial Hospital, Abington, PA USA

**Keywords:** Vantictumab, Nab-paclitaxel, Gemcitabine, Metastatic pancreatic adenocarcinoma, Phase 1b

## Abstract

Vantictumab is a fully human monoclonal antibody that inhibits Wnt pathway signaling through binding FZD1, 2, 5, 7, and 8 receptors. This phase Ib study evaluated vantictumab in combination with nab-paclitaxel and gemcitabine in patients with untreated metastatic pancreatic adenocarcinoma. Patients received vantictumab at escalating doses in combination with standard dosing of nab-paclitaxel and gemcitabine according to a 3 + 3 design. A total of 31 patients were treated in 5 dosing cohorts. Fragility fractures attributed to vantictumab occurred in 2 patients in Cohort 2 (7 mg/kg every 2 weeks), and this maximum administered dose (MAD) on study was considered unsafe. The dosing schedule was revised to every 4 weeks for Cohorts 3 through 5, with additional bone safety parameters added. Sequential dosing of vantictumab followed by nab-paclitaxel and gemcitabine was also explored. No fragility fractures attributed to vantictumab occurred in these cohorts; pathologic fracture not attributed to vantictumab was documented in 2 patients. The study was ultimately terminated due to concerns around bone-related safety, and thus the maximum tolerated dose (MTD) of the combination was not determined. The MAD of vantictumab according to the revised dosing schedule was 5 mg/kg (*n* = 16).

## Introduction

Despite advances in the treatment of pancreatic adenocarcinoma, this cancer remains the fourth most common cause of cancer death in men and women in the United States [1]. Standard first-line treatment options for metastatic pancreatic cancer include FOLFIRINOX and gemcitabine/nab-paclitaxel. The pivotal study of FOLFIRINOX for first line treatment of metastatic pancreatic cancer demonstrated a significant improvement in median overall survival from 6.8 months with gemcitabine to 11.1 months with FOLFIRINOX (HR 0.57; 95% CI, 0.37–0.59; *p* < 0.0001) [2]. Subsequently, gemcitabine/nab-paclitaxel was shown to improve median overall survival in patients with metastatic pancreatic adenocarcinoma to 8.5 months in the first line setting, as compared with 6.7 months in those treated with gemcitabine alone (HR 0.72; 95% CI, 0.62–0.83; *p* < 0.001) [3].

Erlotinib, a small molecule tyrosine kinase inhibitor of the epidermal growth factor receptor (EGFR) pathway, is the only non-cytotoxic agent approved for the treatment of pancreatic cancer; however, it is seldom utilized due to its marginal benefit in this disease. In the study of erlotinib in combination with gemcitabine in advanced pancreatic adenocarcinoma, median overall survival increased from 5.91 months with gemcitabine alone to 6.24 months in combination with erlotinib (HR 0.82; 95% CI, 0.69 to 0.99; *p* = 0.038) [4]. Additional biologic targeted agents have been evaluated in combination with gemcitabine, including anti-EGFR monoclonal antibody cetuximab [5], vascular endothelial growth factor (VEGF) pathway inhibitors bevacizumab [6] and aflibercept [7], and multi-kinase inhibitors axitinib [8] and sorafenib [9]. Unfortunately, each of these studies failed to demonstrate a significant benefit of addition of a biologic targeted agent to gemcitabine therapy.

Better options are needed for treatment of metastatic pancreatic cancer. One limitation in the success of systemic cytotoxic therapy may be related to the presence of cancer stem cells (CSCs). Cancer stem cells are thought to represent a subpopulation of self-renewing malignant cells that generate the heterogeneous cell lineages of the bulk tumor population [10]. These cells have been shown to be resistant to chemotherapy in multiple tumor types, including pancreatic adenocarcinoma [11]. Based on these concepts, the combination of a stem cell targeting agent with cytotoxic chemotherapy is under evaluation in preclinical and clinical settings.

The Wnt/B-catenin pathway is thought to be important in maintaining both normal and cancer stem cells, and is known to play a key role in bone homeostasis [12–14]. This pathway has also been shown to contribute to pancreatic adenocarcinoma development and maintenance [15]. Vantictumab is a fully human monoclonal antibody that inhibits Wnt pathway signaling through binding 5 of the 10 extracellular Frizzled receptors, FZD1, 2, 5, 7, and 8. In preclinical studies evaluating vantictumab in human tumor xenografts, tumor growth inhibition was observed in various models including 6 of 11 pancreatic cancer models. Furthermore, vantictumab decreased the tumor-initiating cell frequency in limiting dilution studies in pancreatic xenografts, further supporting its effects on cancer stem cells [16]. Additional evaluation in pancreatic cancer patient derived xenograft models demonstrated a synergistic response with the combination of vantictumab and nab-paclitaxel [17]. Vantictumab has been evaluated as a single-agent in a phase 1 clinical trial of patients with advanced solid tumors with adequate tolerance, though with documented effects on bone turnover as an on-target effect of Wnt pathway inhibition [18, 19].

An open-label, phase 1b study of vantictumab in combination with nab-paclitaxel and gemcitabine was performed in patients with untreated metastatic pancreatic adenocarcinoma. The primary objective of this study was to evaluate the safety and tolerability of the combination in order to determine the recommended phase 2 dose.

## Patients and methods

### Patient selection

Patients with histologically documented stage IV ductal adenocarcinoma of the pancreas who had not received prior therapy for the treatment of this disease were eligible to participate. Patients were required to be >18 years old, Eastern Cooperative Oncology Group (ECOG) performance status of 0 or 1, have measurable or evaluable disease per Response Evaluation Criteria in Solid Tumors (RECIST) v1.1, and adequate hematologic, hepatic and renal function. Given the target of vantictumab and the anticipated adverse effects associated with this drug related to bone fracture risk, the following patients were excluded: those with osteoporosis by DEXA scan, bone metastases and prior pathologic fracture or lytic lesion requiring impending orthopedic intervention, treatment with thiazolidinedione PPAR gamma inhibitor or long-term glucocorticoid, fasting B-C-terminal telopeptide (B-CTX, a bone turnover marker) of >1000 pg/mL, and history of metabolic bone disease.

The protocol was approved by the local institutional review board. All patients signed a written consent prior to enrollment according to federal and institutional guidelines.

### Study design

Eligible patients were treated with the combination of nab-paclitaxel, gemcitabine and vantictumab by intravenous (IV) infusion in 28-day cycles. Vantictumab was infused prior to nab-paclitaxel and gemcitabine over at least 30 min, with volumes >250 mL administered over at least one hour. Nab-paclitaxel and gemcitabine were then administered at standard doses of 125 mg/m2 and 1000 mg/m2, respectively, on days 1, 8, and 15 of each 28-day cycle.

**Fig. 1:**
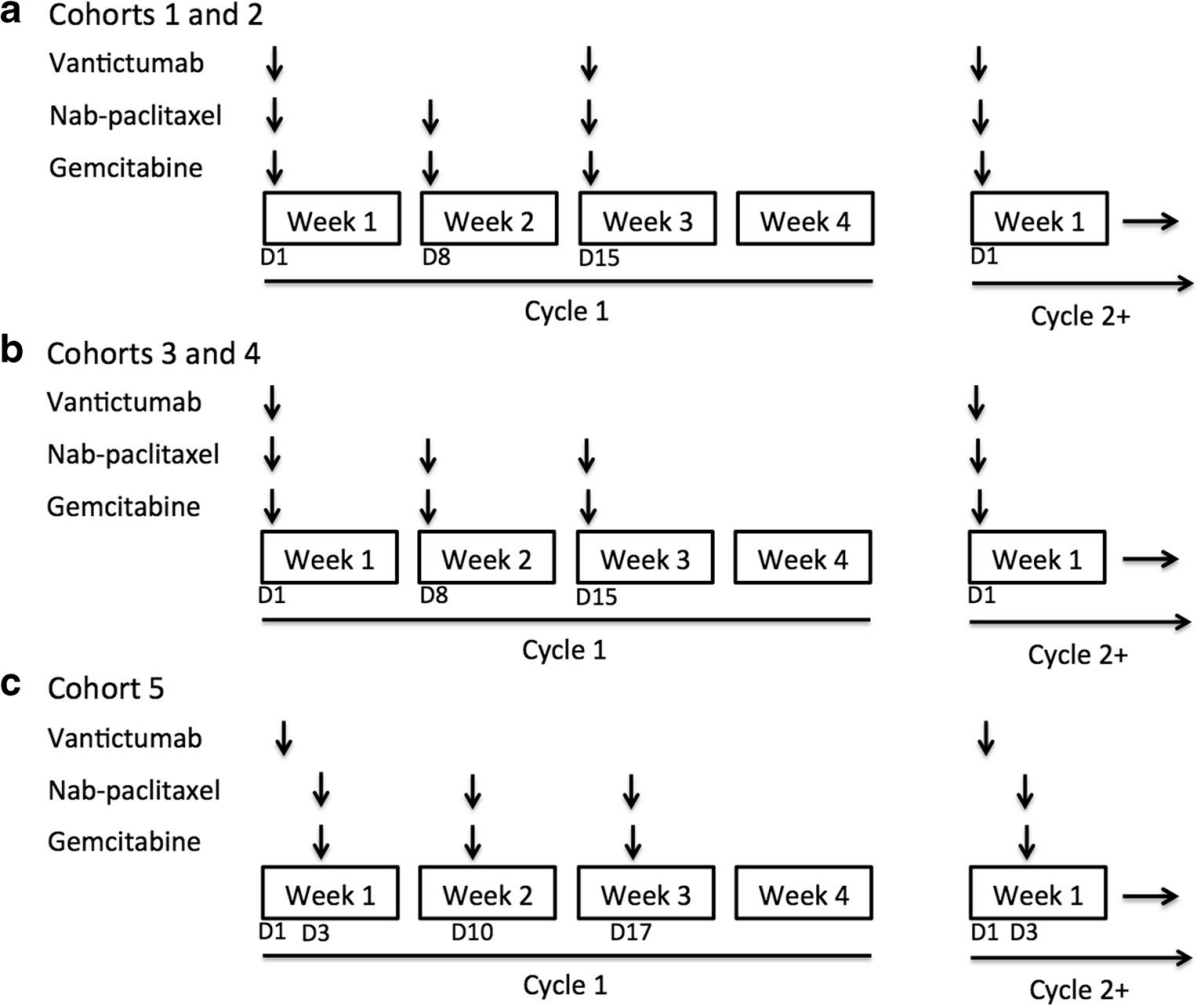
**Treatment schedule across study cohorts a** Treatment schedule for vantictumab every 2 weeks with nab-paclitaxel and gemcitabine weekly for weeks 1–3 in Cohorts 1 and 2 **b** Treatment schedule for vantictumab dosed every 4 weeks with nab-paclitaxel and gemcitabine weekly for weeks 1–3 in Cohorts 3 and 4 **c.** Sequential dosing of vantictumab followed by nab-paclitaxel and gemcitabine in Cohort 5

Initial dosing of vantictumab was determined based on single-agent phase 1 toxicity and pharmacokinetic data [18, 19]. Dosing was started at 3.5 mg/kg IV on days 1 and 15 of each 28-day cycle in Cohort 1 (Fig. 1a). This dose was selected based on the lack of DLT observed in the single-agent vantictumab study at the every 3 week dose levels of 5 mg/kg (same AUC) and 10 mg/kg, as well as the lack of overlap with known toxicity of the nab-paclitaxel and gemcitabine combination.

Due to fragility fractures occurring in Cohort 2 (7 mg/kg every 2 weeks), vantictumab dosing was changed to every 4 weeks for Cohorts 3, 4, and 5. Nab-paclitaxel and gemcitabine dosing remained unchanged (Fig. 1b, c). An initial vantictumab dose of 3 mg/kg was selected for cohort 3, based on a predicted drug exposure less than half of that at the lowest dose at which a fragility fracture was observed across vantictumab trials (5 mg/kg every 3 weeks) in order to minimize risk.

Sequential dosing of vantictumab followed by nab-paclitaxel and gemcitabine was explored in Cohort 5, based on preclinical data demonstrating improved anti-tumor activity when Wnt inhibitors were dosed prior to taxanes [17]. In this cohort, vantictumab was administered day 1 of each 28-day cycle, with nab-paclitaxel and gemcitabine administered days 3, 10, and 17 at standard doses (Fig. 1c).

Initial dose escalation was performed according to a standard 3 + 3 design. A minimum of 3 patients were evaluated at each dose level, with close monitoring for dose-limiting toxicity (DLT) in the first cycle (28 days) of treatment. The maximum tolerated dose (MTD) was defined as the highest dose at which <1 of a maximum of 6 patients experience a treatment-related DLT.

Dose-limiting toxicity was defined as any of the following clinically significant toxicities considered at least possibly related to vantictumab: grade > 4 neutropenia or thrombocytopenia lasting >7 days, > grade 3 febrile neutropenia, > grade 3 increase in total bilirubin or transaminases (unless elevated at baseline), and any > grade 3 non-hematologic toxicity excluding grade 3 electrolyte disturbances responding to correction within 3 days, grade 3 infusion-related reaction resolving to grade < 1 within 24 h without need for hospitalization, grade 3 fatigue lasting less than 4 days after maximum supportive care, and grade 3 nausea, vomiting, diarrhea, rash, and mucositis responding to supportive care within 2 days. Toxicity was graded according to the National Cancer Institute Common Terminology Criteria for Adverse Effects (NCI CTCAE) version 4.03.

Upon protocol modification due to concern for bone toxicity after closure of Cohort 2, dose escalation rules were changed to include a minimum of 6 patients per cohort. The MTD was modified to be the highest dose at which <1 of 6 patients experienced a grade > 1 fragility fracture related to vantictumab, and a bone safety window of 56 days following first administration of study drug was implemented (59 days for staggered dosing in Cohort 5).

Imaging was performed at screening and every other cycle of therapy (approximately every 8 weeks) with assessment according to RECIST v1.1. Levels of tumor marker CA 19–9 were also assessed at these time points.

### Bone safety procedures

Given the known risk for bone toxicity related to Wnt pathway inhibition, and experience in preclinical and single-agent vantictumab studies, bone safety procedures were included in the initial treatment plan. Bone turnover markers including osteocalcin, bone-specific alkaline phosphatase, procollagen type 1 amino-terminal propeptide (P1NP) and B-CTX were obtained after a > 6 h fast between day 22 and 28 of every treatment cycle. DEXA scan for assessment of bone mineral density was also performed between days 22 and 28 of each cycle, starting with cycle 2.

These bone safety procedures also included the initiation of zolendronic acid 5 mg on or before Day 1 of Cycle 1 and then once every 12 months while on vantictumab in the following high-risk patients: 1) patients with a 10-year fracture risk of >20% for any bone or > 3% for the hip according to the FRAX tool at screening and 2) patients with history of fragility fracture of the hip or symptomatic vertebral fracture. In addition, for patients with a B-CTX increase of at least 50% or T-score decline to <−2.5 as per DEXA scan during study treatment, zolendronic acid therapy was initiated. All patients received oral vitamin D and calcium supplementation starting day 1 of treatment through 30 days after discontinuation of vantictumab or 12 months after last dose of zolendronic acid. Vantictumab was discontinued in patients for which B-CTX did not respond to zolendronic acid therapy or bone mineral density continued to decline despite such therapy, and in those who experienced a vantictumab-related fragility fracture.

This bone safety plan was enhanced following the occurrence of bone-related toxicity on study, with 8 bone-related exclusion criteria added and a requirement for postmenopausal females to receive preventative zolendronic acid. In addition, more frequent B-CTX monitoring and the addition of P1NP monitoring were mandated in the first 2 cycles of therapy, with initiation of zolendronic acid and holding or discontinuation of vantictumab according to specific guidelines. Parathyroid hormone (PTH) monitoring with referral to a bone health specialist for elevated levels was also required.

### Pharmacokinetic, Pharmacodynamic, and immunogenicity assessments

Blood samples for assessment of vantictumab pharmacokinetics were obtained at predetermined time points in cycles 1 through 5 and day 1 of every other cycle thereafter in addition to the treatment termination visit. Pharmacodynamic (PD) testing of blood samples was performed in cycles 1 through 3, and at the treatment termination visit. Hair samples for PD assessment were performed in cycles 1 and 2. Anti-drug antibody testing was performed in cycle 1 and every other cycle thereafter, in addition to the treatment termination visit. Optional tumor biopsy samples were collected cycle 1 day 1 and cycle 2 day 8.

### Statistical methods

In this phase 1 study, statistics were descriptive in nature, with no formal statistical hypothesis tested. Statistics were reported using summary tables and listings by dose group and overall patient population. Statistics describing time-to-event variables utilized the Kaplan-Meier method. All analyses were performed using Statistical Analysis Software (SAS) version 9.2 or higher.

## Results

### Patient characteristics

A total of 31 patients were enrolled across 5 sites, and participated in study procedures between November 2013 and May 2017. Relevant characteristics are noted in Table 1. One patient (Cohort 5) who did not meet inclusion criteria related to a thyroid stimulating hormone level below normal limits was enrolled on study. This patient was included in data analysis. All patients received at least one dose of vantictumab, nab-paclitaxel, and gemcitabine.

**Table 1:** Patient demographics and clinical characteristics

Characteristic	Number of patients (%), *N* = 31
Age, years	
Median	66
Range	45–76
Sex	
Male	16 (51.6%)
Female	15 (48.4%)
ECOG performance status	
0	12 (38.7%)
1	18 (58.1%)
Not recorded	1 (3.2%)
Stage at initial diagnosis	
I	0
II	2 (6.5%)
III	1 (3.2%)
IV	26 (83.9%)
Unknown	2 (6.5%)
Previous treatment	
Surgery	3 (9.7%)

### Dose escalation

A total of 3 patients were enrolled in Cohort 1 and received vantictumab 3.5 mg/kg every 2 weeks in combination with nab-paclitaxel and gemcitabine with no DLT observed. The dose was escalated to 7 mg/kg every 2 weeks in Cohort 2. This cohort was expanded beyond 3 patients due to fragility fractures occurring in 2 patients outside of the DLT window. A total of 5 patients were treated at this dose level prior to sponsor-initiated discontinuation of treatment in all patients in Cohorts 1 and 2 related to fragility fractures occurring in this and other ongoing studies of vantictumab.

Following study hold and protocol modification, treatment resumed with Cohort 3. A total of 7 patients were treated with 3 mg/kg every 4 weeks in this cohort, with no DLT identified according to new study criteria and no fractures documented. The dose was thus escalated to 5 mg/kg every 4 weeks. A total of 9 patients were treated at this dose in Cohort 4, with one DLT of grade 3 dehydration documented. One patient experienced a pathologic fracture, not attributed to vantictumab, in this cohort.

Cohort 5 examined sequential dosing of vantictumab followed by nab-paclitaxel/gemcitabine [17]. The same 5 mg/kg every 4 week dose of vantictumab was evaluated. Of the 7 patients in this cohort, no DLT was observed, though an additional patient did experience a pathologic fracture, not attributed to vantictumab.

A summary of treatment received on study is provided in Table 2.

**Table 2:** Treatment on study

Cohort	Vantictumab dose	No. patients treated	No. months treated, median	No. patients with DLT	No. patients with bone fractures
1	3.5 mg/kg q2w	3	3.3	0	0
2	7.0 mg/kg q2w	5	0.6	0	2 (F)^b^
3	3.0 mg/kg q4w	7	7	0	0
4	5.0 mg/kg q4w	9	3.3	1 (Gr 3 dehydration)	1 (P)^c^
5	5.0 mg/kg q4w^a^	7	5.6	0	1 (P)^d^

*F* fragility fracture, attributed to vantictumab; *P* = pathologic fracture, not attributed to vantictumab.

^a^Sequential dosing

^b^T7 compression fracture and sternal fracture in one patient, L1 vertebral fracture in the second

^c^Pelvic fracture

^d^T12 vertebral fracture

### Maximum tolerated dose and maximum administered dose

Grade 3 dehydration attributed to vantictumab, nab-paclitaxel, and gemcitabine in a patient in Cohort 4 was the only DLT occurring on study.

The maximum administered dose in this study was 7 mg/kg every 2 weeks in cohort 2. Given documented fragility fractures in patients on this and other similar vantictumab studies, this dose and schedule was considered unsafe.

After the dosing schedule was revised, the maximum administered dose was 5 mg/kg every 4 weeks. A total of 16 patients were treated at this dose between the standard and sequential dosing schedules. No fragility fractures occurred in these 16 patients, though 2 patients did have documented pathologic fractures related to bone metastases. As the study was ultimately discontinued by the sponsor, the MTD was not determined.

### Toxicities

All patients reported at least one adverse event (AE) while on study. Twenty-nine patients (93.5%) reported AEs related to some component of study treatment (nab-paclitaxel, gemcitabine, or vantictumab), while twenty-six patients (83.9%) reported an AE related to vantictumab.

Of the vantictumab-related AEs, the most commonly reported were nausea, fatigue, dysgeusia, vomiting, constipation and diarrhea (Table 3). Nine patients (29.0%) reported at least 1 vantictumab-related AE of Grade 3 or greater severity. Of these severe AEs, those occurring in more than one patient include fatigue (3 patients, 9.7%), and anemia, thrombocytopenia, dehydration, hypophosphatemia, and nausea (2 patients each, 6.5%). Protocol defined serious adverse events (SAE) attributed to vantictumab occurred in 2 patients, and included grade 3 dehydration in one patient, and grade 3 asthenia, dyspnea, hypothyroidism, and acute renal failure in another patient, all of which resolved.

**Table 3:** Treatment-related adverse events occurring in at least 10% of patients

	Dose Escalation				Sequential Dosing	
Term	3.5 mg/kg q2w (N = 3)	7.0 mg/kg q2w (*N* = 5)	3.0 mg/kg q4w (*N* = 7)	5.0 mg/kg q4w (*N* = 9)	5.0 mg/kg q4w (N = 7)	Overall (N = 31)
**Vantictumab**						
Patients with >1 vantictumab AE	3 (100%)	5 (100%)	3 (42.9%)	8 (88.9%)	7 (100%)	26 (83.9%)
Nausea	1 (33.3%)	3 (60%)	0	2 (22.2%)	5 (71.4%)	11 (35.5%)
Fatigue	1 (33.3%)	1 (20%)	1 (14.3%)	1 (11.1%)	3 (42.9%)	7 (22.6%)
Dysgeusia	1 (33.3%)	2 (40%)	0	2 (22.2%)	1 (14.3%)	6 (19.4%)
Vomiting	0	0	0	1 (11.1%)	5 (74.1%)	6 (19.4%)
Constipation	2 (66.7%)	0	0	0	3 (42.9%)	5 (16.1%)
Diarrhea	0	0	1 (14.3%)	0	4 (57.1%)	5 (16.1%)
Anemia	1 (33.3%)	0	0	2 (22.2%)	1 (14.3%)	4 (12.9%)
Decreased appetite	0	1 (20%)	0	1 (11.1%)	2 (28.6%)	4 (12.9%)
Bone Fracture	0	2 (40%)^a^	0	1 (11.1%)^b^	1 (14.3%)^b^	4 (12.9%)
**Any study component**						
Patients with >1 study treatment AE	3 (100%)	5 (100%)	6 (85.7%)	8 (88.9%)	7 (100%)	29 (93.5%)
Nausea	2 (66.7%)	5 (100%)	4 (57.1%)	4 (44.4%)	6 (85.7%)	21 (67.7%)
Fatigue	3 (100%)	3 (60%)	2 (28.6%)	5 (55.6%)	3 (42.9%)	16 (51.6%)
Anemia	1 (33.3%)	4 (80%)	3 (42.9%)	4 (44.4%)	3 (42.9%)	15 (48.4%)
Alopecia	3 (100%)	2 (40%)	2 (28.6%)	3 (33.3%)	4 (57.1%)	14 (45.2%)
Low platelets	2 (66.7%)	1 (20%)	4 (57.1%)	4 (44.4%)	2 (28.6%)	13 (41.9%)
Neuropathy	1 (33.3%)	2 (40%)	1 (14.3%)	4 (44.4%)	4 (57.1%)	12 (38.7%)
Vomiting	0	1 (20%)	2 (28.6%)	3 (33.3%)	5 (71.4%)	11 (35.5%)
Rash	1 (33.3%)	2 (40%)	3 (42.9%)	2 (22.2%)	1 (14.3%)	9 (29%)
Diarrhea	0	0	2 (28.6%)	2 (22.2%)	4 (57.1%)	8 (25.8%)
Neutropenia	1 (33.3%)	2 (40%)	2 (28.6%)	2 (22.2%)	1 (14.3%)	8 (25.8%)
Decreased appetite	1 (33.3%)	1 (20%)	1 (14.3%)	1 (11.1%)	3 (42.9%)	7 (22.6%)
Dysgeusia	1 (33.3%)	2 (40%	0	2 (22.2%)	1 (14.3%)	6 (19.4%)
Pyrexia	0	3 (60%)	1 (14.3%)	1 (11.1%)	1 (14.3%)	6 (19.4%)
Constipation	2 (66.7%)	0	0	0	3 (42.9%)	5 (16.1%)
Dehydration	0	1 (20%)	1 (14.3%)	1 (11.1%)	2 (28.6%)	5 (16.1%)
Myalgia	0	1 (20%)	1 (14.3%)	0	3 (42.9%)	5 (16.1%)
Pruritus	1 (33.3%)	0	1 (14.3%)	1 (11.1%)	2 (28.6%)	5 (16.1%)
Mucosal inflammation	0	2 (40%)	0	1 (11.1%)	1 (14.3%)	4 (12.9%)

^a^Fragility fractures, attributed to vantictumab

^b^Pathologic fracture, not attributed to vantictumab

Reported adverse events related to any component of study treatment included the addition of anemia, alopecia, thrombocytopenia, neuropathy, rash, neutropenia, decreased appetite and fever to the most frequently documented vantictumab AEs (Table 3). Adverse events of at least Grade 3 severity related to any component of study treatment were documented in 23 patients (74.2%). These severe AEs included neutropenia and fatigue in 6 patients each (19.4%), nausea in 5 patients (16.1%), dehydration in 4 patients (12.9%), anemia, thrombocytopenia, and decreased neutrophil count in 3 patients each (9.7%), and febrile neutropenia and hypophosphatemia in 2 patients each (6.5%).

Bone related adverse events occurred in 4 patients on study. Two of these patients experienced fragility fractures related to treatment in Cohort 2 (7 mg/kg every 2 weeks). These included a Grade 2 T7 compression fracture and sternal fracture in one patient, and a Grade 2 L1 vertebral fracture in a second patient. Pathologic fractures occurred in 2 patients, including a Grade 3 pelvic fracture in a patient in Cohort 4 (5 mg/kg every 4 weeks) who received zolendronic acid at study start related to post-menopausal status. A pathologic Grade 2 T12 fracture was documented in a patient in Cohort 5 (5 mg/kg every 4 weeks, sequential dosing) who received zolendronic acid at study start related to baseline bone metastases. The fragility fractures were attributed to vantictumab therapy while the pathologic fractures were not.

Vantictumab was discontinued related to adverse events in 6 patients, though the AE was attributed to vantictumab in only one of these patients.

### Bone safety assessments

The mean maximum percent change in B-CTX from baseline was a 44.4% (64.24 SD) increase. The largest mean percentage increase occurred in cohort 1 (106.0%, 82.16 SD). In the two patients who experienced fragility fractures in Cohort 2, B-CTX was elevated >50% from baseline to 842 pg/ml and 1321 pg/mL, respectively. Though decreases in P1NP were observed in both patients, these decreases did not meet protocol criteria of decrease of >50% from baseline. In patients with pathologic fractures in the setting of underlying bone metastases, neither increases in B-CTX nor decreases in P1NP met the >50% change threshold.

On DEXA monitoring, one patient on study developed a decline in T-score consistent with osteopenia (from <−1 to > − 2.5), and one patient developed a decline in T-score consistent with osteoporosis (<−2.5). In the later patient, the decline in T-score was only from a baseline −2.4 to an on-study value of −2.5. Notably, the patient experiencing this T-score decline is the same patient from Cohort 4 who developed a pathologic pelvic fracture.

The mean percentage change in FRAX score from baseline at treatment termination was 6.66% (16.12 SD) for any bone, and 20.21% (76.19 SD) for the hip.

A total of twenty-five patients (80.6%) received zolendronic acid on study. Five patients from the first two cohorts (62.5%) received such therapy according to the original bone safety plan, while 20 patients from the subsequent cohorts (87.0%) were treated according to the revised bone safety plan. The most common reason for initiation of bone protective therapy was baseline risk factors according to the revised bone safety plan, followed by increased B-CTX on study, decreased P1NP on study, fracture on study, and decline in DEXA T-score on study. Some patients received zolendronic acid for more than 1 indication.

### Pharmacokinetics and immunogenicity

Vantictumab concentrations were within expected drug exposure levels, with a linear pharmacokinetic (PK) profile and terminal half-life of 4 days. Combination with gemcitabine and nab-paclitaxel did not significantly alter the drug PK. Modest drug accumulation was seen in a fraction of patients.

Only 1 of 31 patients developed anti-drug antibodies, with an incidence of at least 3.2% (not all patient samples were analyzed). The positive test was documented at treatment termination visit, and no associated effect on drug exposure was noted.

### Time on treatment

The mean number of vantictumab infusions was 5.3 (SD; 3.19) and mean duration of treatment per patient was 114.9 days (SD; 92.95). The mean number of nab-paclitaxel infusions administered per patient was 11.3 (SD 8.47) and mean duration of treatment per patient was 122.9 days (SD 99.13). The mean number of gemcitabine infusions administered per patient was 12.7 (SD 9.28) and mean duration of treatment per patient was 137.5 days (SD 103.33).

The most common reason for study discontinuation was disease progression in 16 (51.6%) patients. Of the remaining patients, 5 (16.1%) discontinued due to adverse event, 4 (12.9%) due to patient decision, 3 (9.7%) due to study termination by sponsor, 2 (6.5%) due to clinical progression, and 1 (3.2%) due to investigator decision.

### Antitumor activity

**Fig. 2:**
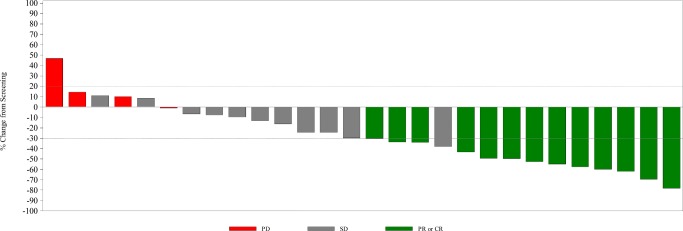
**Waterfall plot of maximum percent change in tumor size across all cohorts** Response documented according to RECIST v1.1 in the intent to treat population per investigator assessment. Progressive disease (PD) is indicated by red bars, stable disease (SD) is indicated by gray bars, and partial response (PR) or complete response (CR) is indicated by green bars

In the intent to treat population, 13 patients (41.9%) had partial response (PR) as best response during study per RECIST v1.1. In addition, 11 patients (35.5%) had stable disease (SD), 4 patients (12.9%) had progressive disease, and 3 patients (9.7%) were not evaluable for response (Fig. 2). This resulted in an overall response rate of 41.9% (95% CI 24.5, 60.9) and clinical benefit rate of 77.4% (95% CI 58.9, 90.4). Decreases in CA 19–9 tumor marker levels were observed in all patients with PR by imaging, with the exception of 2 patients who had normal CA 19–9 levels at baseline. Decreases in CA 19–9 were also observed in 9 of 11 (81.8%) patients with SD on imaging. In patients with PD, an increase in CA 19–9 was observed in 2 of 4 (50%), a decrease was observed in 1 (25%), and the level was not repeated in 1 patient.

### Pharmacodynamics

**Fig. 3:**
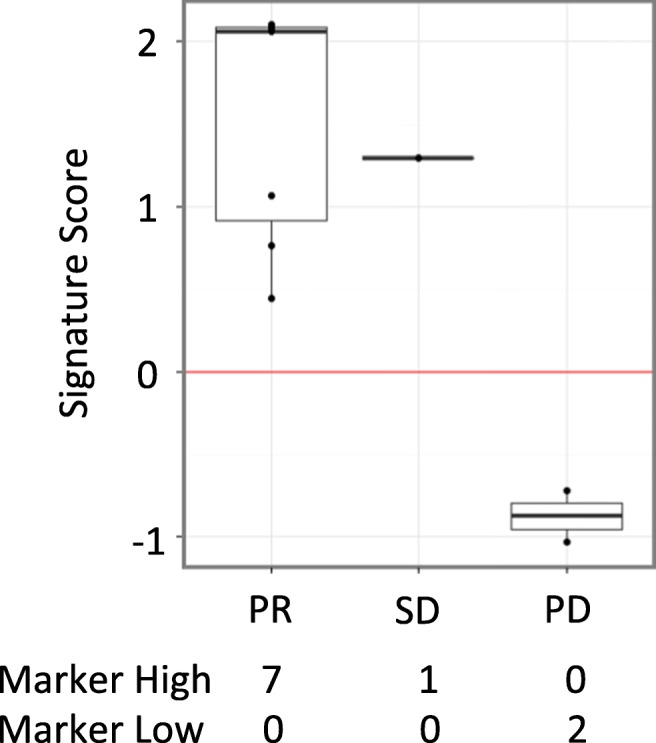
**Distribution of 3-gene signature by best overall response** A total of 10 baseline FFPE tissues were evaluated for the 3 gene biomarker. In this sample of patients, low biomarker signature scores were noted in patients with progressive disease (PD), while higher scores were documented in patients with partial response (PR) and stable disease (SD)

A potential biomarker predictive of response to vantictumab was evaluated in this trial. This 3-gene predictive biomarker (TGFB3, IGF2, and SMO) was found to correlate with tumor growth inhibition in preclinical patient-derived pancreatic cancer xenograft models [20]. In the clinical study, this biomarker was evaluated in baseline tissues from 10 patients, with a significant association noted between a high biomarker gene signature score and best overall response by imaging (*p* = 0.036) (Fig. 3).

### Survival outcomes

Median progression free survival (PFS) in the intention to treat (IIT) patient population was 166.0 days (95% CI 127.0, 270.0), with a range of 1–419 days. Median overall survival (OS) in the IIT population was 305.0 days (95% CI 211.0, 365.0), with a range of 59 to 576 days. The 6-month OS rate was 80.0% (95% CI 60.8, 90.5) and the 12-month OS rate was 30.2% (95% CI 14.0, 48.2).

## Discussion

This phase 1b study of vantictumab in combination with nab-paclitaxel and gemcitabine for the treatment of metastatic pancreatic adenocarcinoma in the front-line setting was discontinued early due to a lack of therapeutic index for vantictumab across multiple studies, with an unacceptable rate of bone fracture. Given the early closure of this trial, the MTD was not determined. However, toxicity data on the 16 patients treated at the 5 mg/kg every 4 week dose level suggests this maximum administered dose at the revised every 4 week schedule to be tolerable. Other toxicity was not significantly increased beyond that expected of nab-paclitaxel and gemcitabine chemotherapy in this trial.

The Wnt signaling pathway is known to play an important role in bone homeostasis [14] in addition to its roles in the development and evolution of multiple malignancies [21]. Bone toxicity was identified in both preclinical and single-agent dose-escalation studies of vantictumab [18]. Despite an integrated plan for reducing risk of bone toxicity in this clinical trial through appropriate selection of subjects, monitoring with bone turnover makers and bone density scans, and initiation of bisphosphonate therapy for high-risk patients, the study was temporarily halted at Cohort 2 of dose escalation related to fragility fractures occurring in this and other vantictumab studies. In this study, no fragility fractures were documented following transition to every 4 week dosing and escalation of the bone safety program in Cohort 3 and beyond; however, two patients had documented pathologic fractures despite treatment with bisphosphonate therapy upon study initiation. Though these were not considered DLT events per protocol, they remain significant in light of the overall toxicity profile of this drug.

Preliminary antitumor activity in this phase 1b study is of interest, with partial response documented in 41.9% and stable disease in 35.5% of patients. The randomized phase 3 study of gemcitabine/nab-paclitaxel versus gemcitabine in metastatic pancreatic adenocarcinoma documented partial response in 23% and stable disease in 27% of patients treated with combination therapy [3]. Median PFS was similar in the study population (166 days, approximately 5.5 months) as compared to the gemcitabine/nab-paclitaxel phase 3 trial (5.5 months). A median OS of approximately 10.2 months (305 days) was documented in the study population, as compared to 8.5 months in the gemcitabine/nab-paclitaxel arm of the phase 3 trial. However, comparison between these studies is clearly limited given the differences in trial design, specifically the small number of highly selected patients and variable vantictumab doses received in this study.

Of additional interest is data demonstrating a significant association between high 3-gene predictive biomarker scores on baseline tumor tissues and response to combination therapy with vantictumab, nab-paclitaxel and gemcitabine. However, this data remains limited by the small number of patients (*n* = 10) assessed in the trial.

A similar study of alternative Wnt pathway targeting agent ipafricept, a recombinant fusion protein comprised of the frizzled family receptor 8 fused to the human immunoglobulin Fc domain, in combination with nab-paclitaxel and gemcitabine for the treatment of metastatic pancreatic adenocarcinoma [22, 23] was also discontinued early for similar reasons, as were combination studies of ipafricept and vantictumab for other tumor types.

Additional agents designed to target cancer stem cells have been investigated in clinical trials for pancreatic cancer. Tarextumab, a fully human antibody that targets cancer stem cells through inhibiting Notch 2 and 3 receptors, was evaluated in a randomized phase 2 trial of 177 patients with metastatic pancreatic adenocarcinoma. These patients received nab-paclitaxel and gemcitabine in combination with tarextumab or placebo in the first line setting. The addition of tarextumab to nab-paclitaxel and gemcitabine did not improve overall survival, but did increase toxicity, and thus the trial was discontinued prematurely [24].

Despite these negative results, interest in targeting the Wnt pathway continues. Multiple porcupine inhibitors, which prevent palmitoylation and secretion of Wnt ligands and thus prevent Wnt activation [25], are now being evaluated in clinical trials. Both LGK974 and CGX1321 are being evaluated alone and in combination with a PD-1 targeting agent in phase 1 dose escalation studies (NCT01351103, NCT02675946), and ETC-1922159 is being evaluated as a single-agent in a phase 1 trial (NCT02521844). Additional agents including Wnt-5a mimic hexapeptide foxy-5, Dickkopf-1-neutralizing monoclonal antibody DKN-01, and small molecule Wnt pathway inhibitor SM08502, among others, are also being evaluated in early clinical trials. Furthermore, approved medications known to target the Wnt pathway, including cyclosporin A, a non-canonical Wnt pathway modulator, are also being evaluated in combination with other pathway modulating agents [26].

Though there is promise in targeting the Wnt pathway in combination with chemotherapy for the treatment of pancreatic adenocarcinoma, future attempts must mitigate the risk of bone-related toxicity. Better understanding of the specific components of the complex Wnt signaling pathway that are key in pancreatic cancer tumorigenesis as well as bone homeostasis will be vital in the development of such treatments.
